# The analysis of *para*-cresol production and tolerance in *Clostridium difficile *027 and 012 strains

**DOI:** 10.1186/1471-2180-11-86

**Published:** 2011-04-28

**Authors:** Lisa F Dawson, Elizabeth H Donahue, Stephen T Cartman, Richard H Barton, Jake Bundy, Ruth McNerney, Nigel P Minton, Brendan W Wren

**Affiliations:** 1 Department of Infectious & Tropical Diseases, London School of Hygiene and Tropical Medicine, Keppel Street, London, WC1E 7HT, UK; 2 Institute of Infection, Immunity and Inflammation, School of Molecular Medical Science, University of Nottingham, Nottingham, NG7 2UH UK; 3 Imperial College London, South Kensington, London, SW7 2AZ, UK; 4 Centre for Integrated Systems Biology, Imperial College (CISBIC), South Kensington, London, SW7 2AZ, UK

## Abstract

**Background:**

*Clostridium difficile *is the major cause of antibiotic associated diarrhoea and in recent years its increased prevalence has been linked to the emergence of hypervirulent clones such as the PCR-ribotype 027. Characteristically, *C. difficile *infection (CDI) occurs after treatment with broad-spectrum antibiotics, which disrupt the normal gut microflora and allow *C. difficile *to flourish. One of the relatively unique features of *C. difficile *is its ability to ferment tyrosine to *para*-cresol via the intermediate *para*-hydroxyphenylacetate (*p-*HPA). *P*-cresol is a phenolic compound with bacteriostatic properties which *C. difficile *can tolerate and may provide the organism with a competitive advantage over other gut microflora, enabling it to proliferate and cause CDI. It has been proposed that the *hpdBCA *operon, rarely found in other gut microflora, encodes the enzymes responsible for the conversion of *p-*HPA to *p*-cresol.

**Results:**

We show that the PCR-ribotype 027 strain R20291 quantitatively produced more *p*-cresol *in-vitro *and was significantly more tolerant to *p*-cresol than the sequenced strain 630 (PCR-ribotype 012). Tyrosine conversion to *p*-HPA was only observed under certain conditions. We constructed gene inactivation mutants in the *hpdBCA *operon in strains R20291 and 630Δ*erm *which curtails their ability to produce *p*-cresol, confirming the role of these genes in *p-*cresol production. The mutants were equally able to tolerate *p*-cresol compared to the respective parent strains, suggesting that tolerance to *p*-cresol is not linked to its production.

**Conclusions:**

*C. difficile *converts tyrosine to *p*-cresol, utilising the *hpdBCA *operon in *C. difficile *strains 630 and R20291. The hypervirulent strain R20291 exhibits increased production of and tolerance to *p-*cresol, which may be a contributory factor to the virulence of this strain and other hypervirulent PCR-ribotype 027 strains.

## Background

*Clostridium difficile *is a spore forming Gram-positive anaerobe and is the leading cause of hospital-acquired diarrhoea worldwide [1,2]. The hospital environment and patients undergoing antibiotic treatment provide a discrete ecosystem where *C. difficile *persists and selected virulent clones thrive. The recent upsurge in the number of *C. difficile *infection (CDI) cases has been linked to the rapid emergence of highly virulent and epidemic strains, known as PCR-ribotype 027. In the UK prior to 2005, 027 strains were rarely reported, but they now cause >33% of the 50,000 cases of CDI reported annually [3]. Several studies have revealed that patients infected with PCR-ribotype 027 strains have more severe diarrhoea, higher mortality and higher level of recurrence [4-8]. This is exemplified by the strain R20291, a prototypical PCR-ribotype 027 strain responsible for the infection of over 160 patients at the Stoke Mandeville hospital, UK in 2004/2005 [9].

CDI characteristically occurs after treatment with broad-spectrum antibiotics. It is thought that antibiotic treatment disrupts the normal gut microflora, providing *C. difficile *with a competitive advantage to colonise the gut mucosa. The reason why *C. difficile *flourish under these conditions is unknown. Following colonisation, toxin production via TcdA and TcdB results in an acute inflammatory-response and severe damage to the intestinal epithelium [10]. These two widely studied toxins are thought to be the main contributors to histopathology and disease burden. However, recent outbreaks of CDI in both Asia and Europe have been attributed to toxin defective (A-B+) strains and are generally PCR-ribotype 017 [11,12]. This suggests that other factors are involved in *C. difficile *pathogenesis, survival and proliferation.

One of the relatively unique properties of *C. difficile *amongst anaerobes is its ability to produce *p*-cresol, a phenolic compound produced by the degradation of tyrosine via *para*-hydroxyphenylacetate (*p-*HPA) [13]. Several studies have shown *p*-cresol is bacteriostatic and inhibits the growth of other bacteria [14]. The production of *p*-cresol by *C. difficile *may provide the bacterium with a competitive advantage over the other gut microflora and facilitate the establishment of the pathogen. Furthermore, the organism itself must have inherent mechanisms to avoid or tolerate the toxic effects of *p*-cresol that may be linked with its ability to produce *p*-cresol. In *C. difficile *it has been hypothesised that *p*-cresol is produced via the oxidation of tyrosine to *p-*HPA followed by the decarboxylation of *p-*HPA to *p*-cresol [15]. However, the temporal production of *p*-cresol and its relative production among different *C. difficile *strains have not been investigated. Genome sequencing of the strain 630 (PCR-ribotype 012) suggests that the *p-*HPA decarboxylase is encoded by three genes (CD0153-CD0155) designated *hpdBCA *[16]. However, the genes involved in the conversion of tyrosine to *p-*HPA are unknown.

In this study we demonstrate the temporal and quantitative production of *p*-cresol by *C. difficile *in both minimal and rich media (supplemented with the intermediate *p-*HPA) using NMR spectroscopy and gas chromatography (zNose™). Gene inactivation mutations in the *hpdA*, *hpdB *and *hpdC *genes in strains 630Δ*erm *and R20291 confirmed the absence of *p*-cresol production in all mutants tested and conclusively show that tyrosine is converted to *p-*HPA by *C. difficile *under minimal media growth conditions. We show that R20291 is more tolerant to *p*-cresol and has a higher capacity to convert tyrosine to *p-*HPA resulting in higher overall levels of *p*-cresol.

## Results

### *Para*-cresol tolerance and production

**Figure 1:**
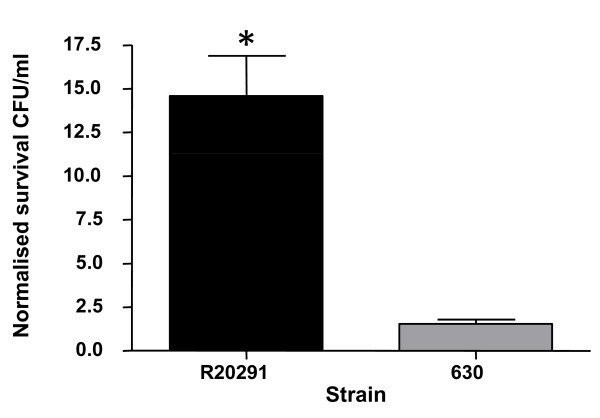
**Tolerance to *p*-cresol**. Strains R20291 and 630 were tested for their *in-vitro *tolerance to 0.1% *p*-cresol. * indicates a significant difference *p *< 0.01 Student's T-test.

The tolerance of strains 630 and R20291 to *p*-cresol was assessed in BHI broth as CFU counts per ml, expressed as a proportion of the untreated control for a four hour incubation period with 0.1% *p*-cresol (Figure 1). Strain R20291 (PCR-ribotype 027) showed a significant increase in survival to 0.1% *p*-cresol compared to strain 630 (PCR-ribotype 012) *p *< 0.01 using a Student's t-test (Figure 1). There was no significant difference in tolerance to *p*-cresol between 630 and 630Δ*erm*, an erythromycin sensitive spontaneous mutant (data not shown). The 630Δ*erm *strain was essential to construct and select gene inactivation mutants for further investigations of *p*-cresol tolerance and production, therefore subsequent analysis was performed with the 630Δ*erm *strain.

**Figure 2:**
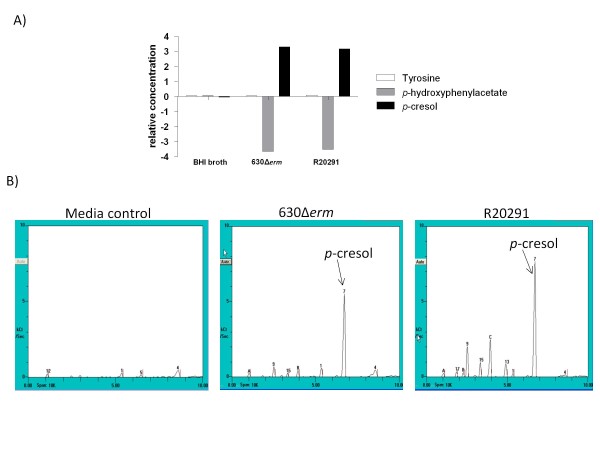
**Detection and production of *p*-cresol by 630Δ*erm *and R20291 using NMR spectroscopy and zNose™ gas chromatography**. The relative production of *p*-cresol in rich media (BHI) supplemented with 0.1% *p-*HPA for strains 630Δ*erm *and R20291 by A) NMR spectroscopy and B) zNose™ gas chromatography. The *p*-cresol peak is indicated with an arrow, at 6.7 seconds for zNose™ experiments

The production of *p*-cresol *in-vitro *was assessed in rich media using two complementary methods, NMR spectroscopy (Figure 2A) and zNose™ (Figure 2B). The production of *p*-cresol was not detected in the *C. difficile *strains 630Δ*erm *or R20291 cultured to stationary phase in rich media (BHI broth, or BHI broth supplemented with cysteine) using either method, despite the availability of tyrosine (data not shown). However, when the strains were grown to stationary phase in rich media supplemented with 0.1% *p-*HPA, *p*-cresol was readily detected by NMR spectroscopy (Figure 2A) and zNose™ (Figure 2B) in both the 630Δ*erm *and R20291 parent strains. The NMR spectroscopy data revealed the conversion of *p-*HPA to *p*-cresol was almost complete, whereas the level of tyrosine remained constant throughout all the samples and the media controls, suggesting that under these conditions the conversion of tyrosine to *p-*HPA was not possible (Figure 2A). The ability of both strains to convert *p-*HPA to *p*-cresol in BHI, yet the inability to convert tyrosine via *p-*HPA to *p*-cresol indicates that constituents in the rich media may inhibit the conversion of tyrosine to *p*-cresol.

### Construction of gene inactivation mutants in *C. difficile*

**Figure 3:**
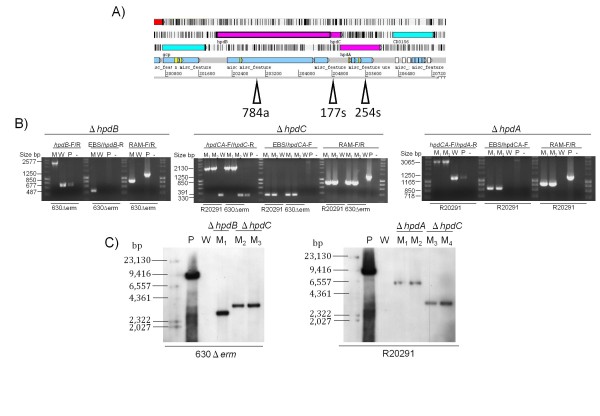
**The *hpdBCA *operon and verification of mutant construction**. The *hpdBCA *operon with insertion sites for the targeted ClosTron mutagenesis, the number refers to the insertion site (bp) and the s/a refers to sense/antisense orientation of the ClosTron insert. B) PCR screen of the mutants (M = mutant; W = wild type; P = plasmid and "-" negative control). Three PCR screens were performed, gene specific forward and reverse primers, intron specific with gene specific primers, and RAM specific primers (Heap et al., 2007). C) Southern blot using a probe specific to the inserted intron. HindIII digests were performed on DNA from M = mutant; W = wild type; P = plasmid. The strains and primer sets are indicated on each figure and in tables 1 and 2. The marker sizes are indicated on the figure and the expected band sizes are as follows for 630Δerm: *hpdB *2.8Kb; *hpdC *3.4 kb and for R20291: *hpdA *- 6.3 kb; *hpdC *- 3.4 kb.

Three co-located genes (*hpdB, hpdC *and *hpdA*) are thought to encode the decarboxylase that converts *p-*HPA to *p*-cresol in strains 630Δ*erm *and R20291. Gene inactivation mutants were constructed using the ClosTron method [17] in strains 630Δ*erm *(mutants 630Δ*erm*Δ*hpdB *and 630Δ*erm*Δ*hpdC) *and strain R20291 (mutants R20291Δ*hpdA *and R20291Δ*hpdC*). The group II intron from the ClosTron system was retargeted using the Sigma TargeTron algorithm to insert into *hpdA*, *hpdB *and *hpdC *in the sense orientation for *hpdA *and *hpdC *at position 254 bp and 174 bp, respectively (from the start of the ORF), and in the antisense orientation for *hpdB *at 748 bp (Figure 3A). Verification of successful mutant construction was performed using three independent PCR screens (Figure 3B). The RAM specific PCR confirmed the loss of the group I intron interrupting the *ermB *RAM, indicating chromosomal integration of the intron. The gene specific and the intron specific primer revealed insertion of the RAM into the target gene, and the gene specific primers flanking the insertion site revealed an increase in 1.9 kb in size for the mutants compared to the wild-type (Figure 3B). The insertion site was verified by sequencing and by Southern blot analysis of the mutants using the intron specific probe which confirmed insertion of a single site-specific group II intron for all the *hpdBCA *operon mutants tested (Figure 3C).

### Analysis of the decarboxylase mutants

**Figure 4:**
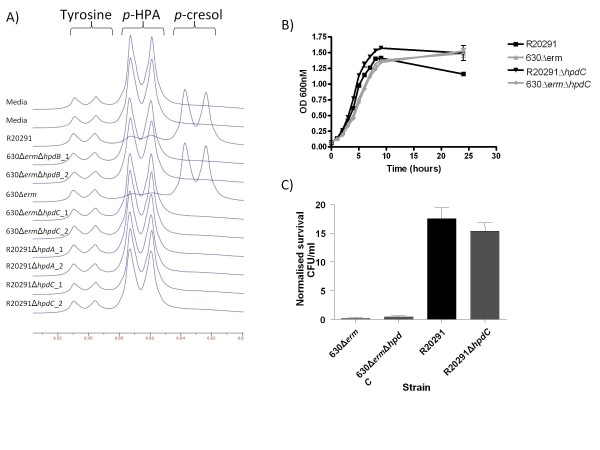
**Analysis of the decarboxylase mutants**. A) NMR spectra showing *p-*cresol production in BHI broth supplemented with 0.1% *p-*HPA for parent and mutant strains, B) Growth curve of the R20291Δ*hpdC *and 630Δ*hpdC *mutants compared to respective parent strains. C) Tolerance to 0.1% *p-*cresol of Δ*hpdC *mutants and respective parent strains.

Initial growth dynamics and NMR spectroscopy analysis revealed that the *hpdB*, *hpdC *and *hpdA *mutants were indistinguishable in terms of the complete lack of *p*-cresol production in rich media supplemented with *p-*HPA (Figure 4A). Subsequent analysis was performed with the *hpdC *mutants as these were constructed in both parent strains R20291 and 630Δ*erm*. Growth curves in minimal media (YP broth) revealed that the R20291Δ*hpdC *mutant grew significantly better than the parent strain R20291, however, no significant difference in *in-vitro *growth was observed between 630Δ*erm*Δ*hpdC *and the respective parent strain (Figure 4B). There were no significant differences between the tolerance of the mutants R20291Δ*hpdC *and 630Δ*erm*Δ*hpdC *to 0.1% *p*-cresol compared to their respective parent strains (Figure 4C), however, the R20291 strains (wild-type and R20291Δ*hpdC*) are significantly more tolerant to *p*-cresol than their 630 counterparts (wild-type and 630Δ*erm*Δ*hpdC) *(*p *< 0.01). The absence of *p*-cresol production observed in the R20291Δ*hpdC *and 630Δ*erm*Δ*hpdC *mutants by NMR spectroscopy in rich media supplemented with 0.1% *p-*HPA (Figure 4A), was reproducible in minimal media using zNose™ gas chromatography (data not shown).

### Temporal production of *p*-HPA and *p*-cresol in mutant and parent strains

**Figure 5:**
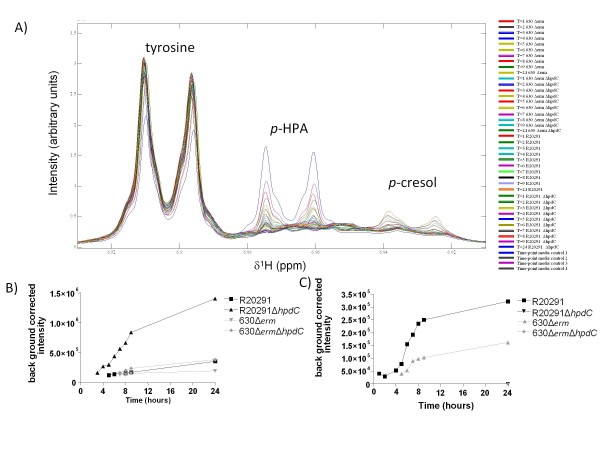
**Temporal production of *p-*HPA and *p*-cresol in mutant and wild-type strains using NMR**. A) NMR spectra showing an overview of the relative levels of tyrosine, *p-*HPA and *p*-cresol from all replicates and strains tested over a 24-hour time period, the colours define the 44 samples used in the time course experiment, over four strains and media controls. T = time of sampling (hours post inoculation). B) The relative production of *p-*HPA by mutant and patent strains over a 24-hour time period. C) The relative production of *p*-cresol by the parent strains over a 24-hour time period. (The levels of *p-*cresol by the Δ*hpdC *mutants were below the limits of detection by NMR and were not plotted).

Preliminary NMR spectroscopy revealed that *p*-cresol was produced in unsupplemented minimal media (YP broth), indicating that the available tyrosine was converted to *p*-cresol via the intermediate *p-*HPA. The temporal production of *p-*HPA and *p*-cresol were assessed in minimal YP media, using both wild-type and mutant strains of R20291 and 630Δ*erm*. For each strain, samples were taken every hour for the first 8 hours with a final time point of 24 hours, after which the relative production of *p-*HPA and *p*-cresol were determined by NMR spectroscopy, the combined data for all the strains and controls is presented in Figure 5A. High levels of tyrosine were present in all samples including the media control (Figure 5A); however, the conversion to *p-*HPA and *p*-cresol across all the strains was limited to a few samples (Figure 5A), namely the latter time points in the parent strains. In the decarboxylase mutants R20291Δ*hpdC *and 630Δ*erm*Δ*hpdC*, a build up of *p-*HPA was evident from 4 to 24 hours (Figure 5B and 5C). The level of *p-*HPA production was significantly higher in the R20291Δ*hpdC *mutant compared to the 630Δ*erm*Δ*hpdC *mutant (Figure 5B and 5C). As predicted, *p*-cresol was not detected in the mutant samples. In the parent strains *p*-cresol production was detected at 4 hours and increased until the levels reached a peak at 24 hours (Figure 5C), but the relative level of *p*-cresol produced was significantly higher in R20291 than 630Δ*erm*.

## Discussion

In this study we show two independent methods for measuring levels of *p*-cresol from *C. difficile *grown *in vitro*. NMR spectroscopy and gas chromatography (zNose™) provide a quantitative means of measuring the relative and temporal production of *p*-cresol by *C. difficile*. This revealed that that *p*-cresol is only produced from the conversion of tyrosine in minimal media. indicating that *p*-cresol production may be linked to the limitation of nutrients, or nutrient stress. However, the successful conversion of *p*-HPA to *p*-cresol in rich media suggests the limiting step in the cascade is the utilisation of tyrosine. Rich media may contain a constituent(s) such as glucose, which inhibits the conversion from tyrosine to *p-*HPA.

Gene inactivation mutations in the *hpdB*, *hpdC *and *hpdA *genes in strains 630Δ*erm *and R20291 revealed the complete absence of *p*-cresol production in all mutants tested, confirming the role of the putative decarboxylase operon in *p*-cresol production in *C. difficile*. The build up of *p-*HPA observed in the *hpdBCA *operon mutants confirm that *C. difficile *converts tyrosine to *p-*HPA, rather than using an exogenous source of *p-*HPA and this conversion is significantly more efficient in R20291. With the exception of *Clostridium scatologenes*, the *hpdBCA *operon appears absent from the genomes of other sequenced anaerobic bacteria [18]. The production *of p*-cresol coupled with its ability to produce tissue-damaging toxins may explain why *C. difficile *is almost unique among pathogens in causing antibiotic associated colitis. The production of *p*-cresol by *C. difficile *may provide a competitive advantage over other microorganisms during re-colonisation of the gut. If this hypothesis is true, *C. difficile *should itself be tolerant to the bacteriostatic properties of *p*-cresol. Previous studies have shown that in contrast to most other anaerobes, *C. difficile *is more tolerant to *p*-cresol [14]. We have also shown that the modern hypervirulent 027 clonal lineage has a propensity to be more tolerant to *p-*cresol than other strains, as observed with BI-16, a recent 027 isolated which exhibits a level of tolerance similar to R20291. The historic 027 isolate CD196 exhibits a similar level of tolerance to strain 630 [18]. This increase in tolerance to *p*-cresol in the modern hypervirulent 027 isolates may be linked to increased virulence.

In addition, the hypervirulent PCR-ribotype 027 strain has a higher capacity to convert tyrosine to *p-*HPA resulting in a higher overall yield of *p*-cresol. Analysis of the decarboxylase mutants revealed that although *C. difficile *can tolerate *p*-cresol, high levels have a deleterious effect on the growth rate of *C. difficile*, as the mutants grow better *in-vitro *than their respective parent strains. Although it is evident that the 027 ribotype R20291 is more tolerant to *p*-cresol and produces significantly more *p*-cresol than other strains, the mechanism of tolerance to *p*-cresol does not appear to be linked to its production. These results indicate that there is an intricate balance between optimal *p*-cresol production and deleterious effects on growth.

## Conclusions

The hypervirulent R20291 strain produces high levels of *p*-cresol, and has an elevated tolerance, which may contribute to the colonisation and dissemination of the 027 clonal lineage by providing a selective advantage. There is a delicate interplay between relative *p*-cresol production and growth rate, whereby R20291 may have reached an advantageous compromise.

## Materials and methods

### Bacterial strains and culture

*C. difficile *strains used in this study were 630, 630Δ*erm *and R20291. Strain 630, PCR-ribotype 012, was originally isolated from a patient with severe PMC in Zurich, Switzerland in 1982. 630Δ*erm *is an erythromycin sensitive strain that was isolated after passage of the original sequenced strain 630 [19]. Erythromycin sensitivity is required for the construction of *C. difficile *gene inactivation mutants. R20291, a hypervirulent PCR-ribotype 027 strain was isolated from an outbreak at Stoke Mandeville hospital in 2006 and was provided by Jon Brazier (Anaerobe reference laboratory, Cardiff, UK). Strains were stored at -80°C and were cultured on BHI Agar (Oxoid), supplemented with 0.05% L-cysteine and cycloserine/cefoxitin antibiotic supplement (Fluka) at the recommended concentrations for 1 to 2 days under anaerobic conditions, in a Modular Atmosphere Control System 500 (Don Whitney Scientific) at 37°C. Liquid cultures were grown in BHI broth (Oxiod) supplemented with 0.05% L-cysteine and cycloserine/cefoxitin antibiotic supplement (Fluka) with and without 0.1% *p-*HPA (Sigma), or in yeast peptone (YP) broth, 16 gL^-1 ^peptone (Sigma), 5 gL^-1 ^yeast (Sigma), and 5 gL^-1 ^NaCl_2 _(Sigma). *E. coli *strain CA434, the conjugation donor, was grown in Luria-Bertani (LB) broth or agar supplemented with 12.5 μg/ml chloramphenicol.

### *Para*-cresol tolerance assays

Primary cultures were inoculated with three single colonies into pre-equilibrated media, shaking at 50 rpm on an orbital shaker. At an OD_600 _nm of 0.3-0.4, liquid cultures were inoculated 1/10 into pre-equilibrated media containing 0.05% (v/v) and 0.1% (v/v) *p*-cresol alongside an untreated control. These were incubated under anaerobic conditions for 4 hours before colony forming units were performed in pre-equilibrated 1 × PBS (Sigma), then plated in triplicate onto BHI plates and incubated for 24 hours under anaerobic conditions. CFU counts were determined for all of the test conditions and were calculated per ml of culture. The *p*-cresol stress CFU data was normalized to the untreated control and expressed as a percentage. Data was analysed in GraphPad Prism V4.02 using a two-tailed Student's t-tests with a *p *value cut off of *p *< 0.01.

### NMR

Primary cultures of *C. difficile *were grown overnight as outlined above in either BHI broth, BHI supplemented with 0.1% *p-*HPA, or YP broth. Secondary cultures were inoculated 1/10 from the primary cultures into the relevant media. Samples were removed every hour up to 24 hours, the OD_600 _nm was taken and samples were double filter sterilized using 0.2 μM filter, then stored at -80°C.

^1^H NMR spectroscopy analysis was carried out to determine the production of *p*-cresol in rich media supplemented with *p-*HPA, and for determination of the temporal production of *p-*HPA and *p*-cresol in the mutant and wild-type strains to yield the relative levels of tyrosine and of the metabolites produced, *p-*HPA and *p*-cresol. Spectra were obtained using buffered extracts of the various cultures. Typically, 350 μl of sample was transferred to a 5 mm Norell HP507 NMR tube, and 150 μl of a pH 7.4 phosphate buffer with TSP added as a chemical shift reference was then added, providing a final sample volume of 500 μl.

All ^1^H NMR spectroscopy was carried out on a Bruker Avance-DRX600 instrument operating at 600.29 MHz, using a 5 mm TXI probe (Bruker BioSpin GmbH, 76287 Rheinstetten, Germany). The standard 1-D pulse sequence [RD-90°-t_1_-90°-t_m_-90°- acquire FID] was employed for all acquisitions, with water peak suppression achieved through irradiation of the water signal during t_m _and RD, using 8 dummy scans, a spectral width of 20.02 ppm, Fourier transform line broadening of 0.3 Hz, t_m _= 150 ms, and t_1 _= 3 μs. The first acquisition program for the rich media samples used 64 scans, 32 k time and frequency domain points, and a relaxation delay (RD) of 3.5 s. The second acquisition run for the temporal analyses employed 128 scans, and used a higher spectral resolution of 64 k time and frequency domain points, with a reduced relaxation delay (RD) of 2.137 s to maintain the across-acquisition quantitation status of the metabolites of interest. Within each run, the instrument receiver gain was set to a constant value for all samples.

The temporal metabolite profile analyses were carried out starting with Matlab R2008a (MathWorks Inc, Natick MA, USA), using proprietary in-house routines for some of the spectral import processing and for correlation analysis. Data was prepared and imported to Matlab initially using MetaSpectra over the spectral region between 0 and ~10.1 ppm, using 32 k data points, which is very close to the original acquisition digitisation density of 64 k over a 20.11 ppm sweep width. No spectral excision for the water residual signal region was made. Under the assumption of constant linewidth, relative quantitation for *p-*HPA and *p*-cresol in this work was based on peak heights for the higher shift peak from each doublet (6.875 and 6.838 ppm). Peak height quantitation under these assumptions has been shown to be a reliable quantitative approach [20]. The TSP peak height and line width for the data array was used to verify this was a reasonable assumption, as well as confirming volumetric accuracy in sample preparation. This quantitation data was then placed into an Excel spreadsheet for calculation of the baseline corrected values, using the local baseline taken from the broth control samples having zero *p-*HPA and *p*-cresol present. Some STOCSY analysis of the data arrays (data not shown) was also used to confirm the conversion pathway sequence, by showing the appropriate anti-correlations in the levels of precursor and conversion metabolite [21]. The metabolite quantitation data was then graphed using GraphPad Prism.

### zNose™

The zNose™ is an ultra rapid analytical device that allows real time monitoring of volatile compounds [22], by combining miniaturised gas chromatograph separation technology with a highly sensitive acoustic wave sensor. Primary and secondary cultures of *C. difficile *were set-up as outlined above and harvested at OD_600_nM 0.4 and at 24 hours, then these were transferred into pre-baked (overnight at 210°C) 40 ml glass vials sealed with screw caps with an integral PTFE/silicone septa (Supelco, Gillingham, UK).

Measurements were performed with a zNose™ Model 7100 bench top vapour analysis system (Electronic Sensor Technology, Newbury Park, CA) fitted with a capillary DB-624 column and a temperature controlled surface acoustic wave (SAW) detector. Headspace samples were withdrawn from the sealed vials via a side hole Luer needle inserted through the septum. Ten second samples were taken at a flow rate of 0.5 ml/second. All measurements were taken at ambient temperature. The column was ramped at from 40°C to 160°C at 10 C/s in a helium flow of 3.00 cm^3^. The SAW sensor operated at a temperature of 60°C and data were collected every 0.02 s. After each data sampling period the sensor was baked for 30 s at 150°C to remove any residual deposit and an air blank was run to ensure cleaning of the system and a stable baseline. On encountering compounds exiting the DB-624 column the SAW detector registers a depression in the frequency of the acoustic wave at its surface relative to a reference sensor. Derivativisation is performed automatically by the Microsense software (EST, Newbury Park, CA) and retention time and peak sizes are plotted. A reference sample of pure *p*-cresol (Sigma) was run under the same conditions to confirm that the retention time of the experimental peak observed (6.7 s) was identical to that of the target compound.

### Production of gene inactivation mutants

The genes of the *hpdBCA *operon were insertionally inactivated using the ClosTron system in strains 630Δ*erm *and R20291 [17]. The group II Ll.LtrB intron was retargeted to *hpdB*, *hpdC*, and *hpdA *by SOEing PCR as previously described [17] with oligonucleotides (listed in Table 1) designed using the Sigma TargeTron website (http://www.sigma-genosys.com/targetron/). PCR products were cloned into pGEM^®^-T Easy (Promega) as outlined in the manufacturer's guidelines to create the plasmids pLDhpdA1 and pLDhpdC1, listed in Table 2. The sequence of the retargeted intron regions were confirmed by sequencing using primers T7 and SP6 with the BigDye^® ^Terminator v3.1 Cycle Sequencing Kit (Applied Biosystems) in accordance with the manufacturer's guidelines.

**Table 1:** List of oligonucleotides used in this study

Oligonucleotide	Sequence
*hpdB*-IBS	AAAAAAGCTTATAATTATCCTTATACCACTAAGCCGTGCGCCCAGATAGGGTG

*hpdB*-EBS1δ	CAGATTGTACAAATGTGGTGATAACAGATAAGTCTAAGCCCATAACTTACCTTTCTTTGT

*hpdB*-EBS2	TGAACGCAAGTTTCTAATTTCGGTTTGGTATCGATAGAGGAAAGTGTCT

*hpdA*-IBS	AAAAAAGCTTATAATTATCCTTAGGTATCGGCAAAGTGCGCCCAGATAGGGTG

*hpdA*-EBS1δ	CAGATTGTACAAATGTGGTGATAACAGATAAGTCGGCAAATGTAACTTACCTTTCTTTG

*hpdA*-EBS2	TGAACGCAAGTTTCTAATTTCGATTATACCTCGATAGTGGAAAGTGTCT

*hpdC*-IBS	AAAAAAGCTTATAATTATCCTTATATGTCATGGTAGTGCGCCCAGATAGGTG

*hpdC*-EBS1δ	CAGATTGTACAAATGTGGTGATAACAGATAAGTCATGGTAAGTAACTTACCTTTCTTTGT

*hpdC*-EBS2	TGAACGCAAGTTTCTAATTTCGGTTACATATCGATAGAGGAAAGTGTCT

EBS universal	CGAAATTAGAAACTTGCGTTCAGTAAAC

*hpdB*-F	AATGCCATGGGTAAGTGAAAGC

*hpdB*-R	GAATTGTATAAGTCAACTGAAGAGC

*hpdCA*-F	GTGGATGCAACCAAAGGAAT

*hpdC*-R	TTACAACTCAGTGGACATCCATT

*hpdA*-R	TTAGAAAGCTGTCTCATGAC

RAM-F	ACGCGTTATATTGATAAAAATAATAATAGTGGG

RAM-R	ACGCGTGCGACTCATAGAATTATTTCCTCCCG

SalI-R1	ATTACTGTGACTGGTTTGCACCACCCTCTTCG

EBS2	TGAACGCAAGTTTCTAATTTCGGTTTGGTATCGATAGAGGAAAGTGTCT

**Table 2:** List of plasmids used in this study

Plasmid	Relevant properties	Source
pGEM^®^-T Easy	Commercial TA' cloning plasmid	Promega

pMTL007	ClosTron mutagenesis plasmid	Heap *et al. *2007

pLDhpdB	pMTL007 carrying Ll.LtrB intron retargetted to *hpdB*	This work

pLDhpdC1	pGEM^®^-T Easy carrying Ll.LtrB intron retargetted to *hpdC*	This work

pLDhpdC2	pMTL007 carrying Ll.LtrB intron retargetted to *hpdC*	This work

pLDhpdA1	pGEM^®^-T Easy carrying Ll.LtrB intron retargetted to *hpdA*	This work

pLDhpdA2	pMTL007 carrying Ll.LtrB intron retargetted to *hpdA*	This work

The retargeted intron was then cloned into the *Hin*dIII and *Bsr*GI sites of pMTL007 to create the plasmids pLDhpdA2, pLDhpdB, and pLDhpdC2 (Table 2), which were transformed into the *E. coli *conjugation donor strain CA434 and transferred into *C. difficile *strains 630Δ*erm *and R20291 by conjugation as previously described [23]. Transconjugants were selected for in the presence of thiamphenicol (15 μg/ml, Sigma), after which mobilisation of the intron from the plasmid to the gene of interest was induced using IPTG. Screening for chromosomal insertion of the intron was performed with lincomycin (20 μg/ml, Sigma) for both *C. difficile *630Δ*erm *and R20291 to select for the restored *ermB *retrotransposition-activated marker (RAM) that signals integration into the genome. DNA was extracted for analysis from colonies, which were phenotypically lincomycin resistant, but thiamphenicol sensitive to indicate loss of the plasmid pMTL007. Potential mutants were verified by PCR, sequencing and Southern blot analysis.

### Screening of mutants by PCR, sequencing and Southern blot

Potential mutants were screened by PCR, sequencing and Southern blot analysis to confirm the chromosomal integration of the intron within the desired genes and loss of the plasmid pMTL007. Three PCRs were performed to screen putative mutants using the following oligonucleotides (Table 1): i) RAM-F and RAM-R, to screen for loss of the group I intron, which insertionally inactivated the *ermB *RAM prior to chromosomal integration of the group II intron; ii) a gene specific primer and the group II intron specific EBS universal primer, to screen for insertion of the intron into the desired location in the genome; and iii) gene specific forward and reverse primers that flank the insertion site. Genomic DNA from *C. difficile *R20291 and 630Δ*erm*, and plasmid DNA from pMTL007 were used as controls for the PCR reactions. PCR reactions were performed with Go*Taq*^® ^PCR mix (Promega) in accordance with the manufacturers guidelines. The thermal cycling conditions were as follows: 95°C for 2 min × 1; 95°C for 30 sec, 50°C for 30 sec, 68°C for 8 min × 35 cycles; and 68°C for 10 min × 1.

Sequencing was performed across the junction of the gene to intron using gene specific primers and the EBS universal primer to verify insertion site. Southern blot analyses were performed using Roche DIG-High Prime DNA labelling and detection reagents, in accordance with the manufacturer's guidelines and visualised using CDP star (Roche). Genomic DNA from wild type and potential mutants was disgested with HindIII alongside plasmid DNA as a positive control. The probe was produced by PCR using SaII-R1 and EBS2 primers (Table 1), designed within the group II intron sequence.

## Authors' contributions

LFD, EHD, STC and NPM helped in the construction and characterisation of mutants. RHB, JB and RM performed spectroscopy and zNose™ analyses. LFD, EHD and BWW wrote the manuscript and BWW conceived the study. All authors read and approved the final manuscript.
